# Evaluation of the whole auditory pathway using high-resolution and functional MRI at 7T parallel-transmit

**DOI:** 10.1371/journal.pone.0254378

**Published:** 2021-09-07

**Authors:** Sandra Da Costa, Jérémie Clément, Rolf Gruetter, Özlem Ipek

**Affiliations:** 1 Centre d’Imagerie Biomédicale, Ecole Polytechnique Fédérale de Lausanne (EPFL), Lausanne, Switzerland; 2 Laboratory for Functional and Metabolic Imaging, Ecole Polytechnique Fédérale de Lausanne (EPFL), Lausanne, Switzerland; 3 School of Biomedical Engineering and Imaging Sciences, King’s College London, London, United Kingdom; New York University Langone Health, UNITED STATES

## Abstract

**Purpose:**

The aim of the present study is to show a MR procedure for the evaluation of simultaneous left and right auditory functions with functional MRI, and high-resolution acquisition of anatomical auditory pathway using parallel-transmit (pTx) methods at 7T.

**Methods:**

The time-efficient MR acquisition included two steps: RF weights were optimized for the regions-of-interest and high-resolution MR images of the inner-ear were acquired for the first 30 min (400 μm-iso resolution) followed by functional MRI acquisitions along the whole auditory pathway during the next 20 minutes. Data was processed with a linear cross-correlation analysis to define frequency preferences for each voxel in the auditory relays.

**Results:**

Tonotopic maps revealed ordered bilateral frequency gradients in the auditory relays whereas at the level of the cochlear nuclei and superior olivary complexes the frequency gradients were less evident. A 21% increase in transmit-field efficiency was achieved over the left/right inner-ear regions and thus its main structures were clearly discernible using the pTx methods, compared to a single transmit RF coil.

**Conclusion:**

Using 7T pTx allows a fast (less than 60 min in total) and qualitative evaluation of the simultaneous left and right auditory response along the entire auditory pathway, together with high-resolution anatomical images of the inner-ear. This could be further used for patient examination at 7T.

## Introduction

Despite a large number of studies investigating the auditory relays in healthy subjects such as the Cochlear Nuclei: [1,2], the Inferior Colliculi: [2], the Medial Geniculate Bodies: [3], the Auditory Cortices: [4–7], or in tinnitus [8,9], little is known on how hearing loss or Menieres’ disease modify functional representations along the human auditory pathway. In a normal subject, the auditory information is decomposed at the level of the cochlea according to its frequency in a specific organization named cochleotopy–or tonotopy–, where low and high frequencies activate hair cells in the center and at the base of the cochlea, respectively.

Due to the increased SNR and spatial resolution at 7T, fMRI studies focusing on each of the auditory relays separately have consistently reported that the cochlear frequency decomposition is conveyed as a clear, specific frequency gradient ranging from low to high frequencies in all the different subcortical auditory structures (collicular nuclei and Superior Olivary Complex: [1], inferior colliculi: [3,10] and medial geniculate bodies: [3]), until the auditory cortices. In hearing impaired humans, the damages concomitant with hearing loss or Meniere’s disease at the level of the cochlea has been reported to induce a broadening of the auditory filters at the cochlear level and then an enlargement of the cortical representations near the “lesion-edge” frequency (i.e. the frequency lost at the cochlear level) [9,11]. However, none of the aforementioned studies above investigated simultaneously the tonotopic gradient reorganization within the other auditory relays. A better understanding of how the frequency representations are modified by hearing loss or Meniere’s disease would help facilitate diagnosis and help the audiologists or the otorhinolaryngologist to orient the patient to an efficient pharmacological or cognitive treatment.

The cochlea, together with the semi-circular ducts (vestibular system) form the inner-ear. MR imaging of this region may contribute to a reliable diagnostic of health deficit [12,13] as for Menieres’ disease [14–17] or hearing loss [18,19]. For example, endolymphatic hydrops, that are a disorder of the inner-ear associated with Meniere’s disease, can be imaged with high contrast with respect to the surrounding tissues using gadolinium-based contrast-agents [14,16,20].

While the aforementioned studies on structural imaging of the inner-ear were performed on clinical MR scanners at 1.5T or 3T, going to ultra-high MR (B_0_ ≥ 7T) leads to an increased signal-to-noise ratio (SNR) [21] and improved spatial resolutions [22]. This potentially allows a more precise depiction of the membranous structures of the inner-ear such as Reissner’s membrane, macula and basilar membrane that are crucial to evaluate the patient’s condition [23–25].

Nevertheless, RF inhomogeneity is more pronounced at 7T due to the shorter wavelength (λ≈ 12 cm in the brain) [26], which is compounded in the inner-ear regions that are located on the bottom and lateral sides of the brain where birdcage coils, typically used at 1.5T or 3T, produce low transmit field efficiency [26,27]. Moreover, the proximity of air-filled areas alters the B_0_ homogeneity and can lead to signal loss at the interface with tissues. To address the RF inhomogeneity, dielectric pads were proposed to improve the transmit field produced by volume coils as the birdcage operated in the default single transmit mode. When placed within the volume of the coil and near the region-of-interest, the RF field can be locally enhanced [28,29]. Such dielectric pads were, for example, used for inner-ear imaging at 7T [25]. However, their dimensions, placement, compounds, and efficiency depend on the subject and the RF coil setup. Therefore, they are usually designed for a specific application, which limits the suitability of a given dielectric pad to diverse MR imaging scenarios.

Alternatively, parallel-transmit (pTx) coil arrays could be used: by manipulating the RF phases and amplitudes of each transmit element in a multi-channel transmit array, constructive transmit (B_1_^+^‐field) interferences can be generated over the region‐of‐interest (ROI) and thus improve the homogeneity of the transmit field [30–32] compared to single channel coils [33]. High-resolution MR images of the inner-ear have been previously reported using pTx coil arrays in combination with dedicated receivers [34]. However, the receive coil setup was optimized to cover specifically the ear regions, excluding the auditory cortices. Using a whole-brain pTx coil array and pTx methods, the RF field may be optimized to investigate during a single scan session the different structures involved in hearing as the inner-ear, the cochlear nuclei, superior olivary complex, inferior colliculi and medial geniculate bodies located at the center of the brain and the auditory cortices situated in the temporal lobes.

Therefore, the aim of this study was to introduce and investigate an MR procedure for parallel-transmit integrating the high-resolution MR imaging of the inner-ear at 7T together with an evaluation of the auditory functional response along the whole auditory pathway, compatible with clinical examination.

## Material and methods

The measurements were performed on a 7T Magnetom MR scanner equipped with 8 × 1 kW RF amplifier (Step 2.3, Siemens, Erlangen, Germany) and 32 receive channels on healthy volunteers (N = 9, age range = 21.87 ± 2.9 years, 4 men and 5 women). This study was approved by the local ethics committee of the Canton de Vaud. All the subjects were recruited on the EPFL campus and all had signed an informed written consent. None reported neurological or psychiatric illness or hearing deficits. All research was performed in accordance with relevant guidelines in the field of ultra-high resolution imaging.

### Parallel-transmit mode

Parallel-transmit MR measurements were performed using an in-house built 8Tx/32Rx dipole coil array geometrically optimized for whole-brain coverage, including the cerebellum and subcortical regions [35,36].

#### RF phase shimming

**Fig 1:**
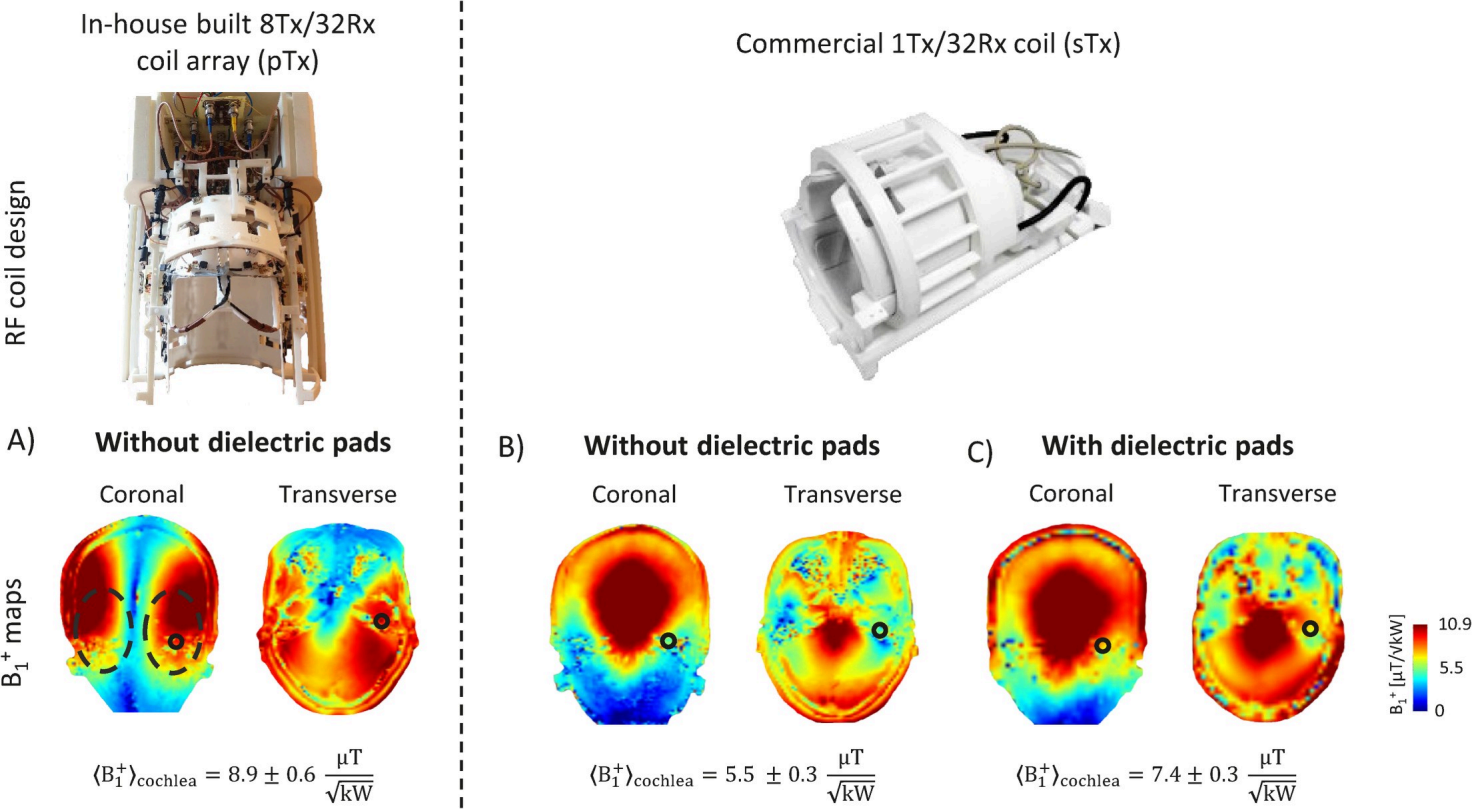
RF coil models and transmit field maps in the inner-ear region. First row: Photos of the 8Tx/32Rx (pTx) coil array (left) and the commercial 1Tx/32Rx (sTx) coil (right). Second row: B_1_^+^ maps shown in coronal and transverse slices centred on inner-ears in: A) pTx mode, B) & C) sTx mode without and with dielectric pads, respectively. In pTx mode, RF phases were optimized over the two lateral sides of the brain (dashed black circles). The mean B_1_^+^ value is given in each situation averaged over a smaller field-of-view encompassing the inner-ear (continuous black circles).

The transmit field was optimized separately for each subject in pTx mode over the cortical areas (auditory cortices) and subcortical nuclei along the auditory pathway for fMRI (inferior-colliculus, medial geniculate bodies and cochlear nuclei). For inner-ear imaging, two ROIs covering the two lateral sides of the brain were selected on a coronal single-slice centred on the position of the inner-ears (Fig 1A). The two regions covered not only the auditory cortices (as shown in [10]) but were extended towards the inner-ear region. The transmit field was optimized simultaneously over the two regions with a cost function that aimed to minimize the root-mean-square difference between the combined transmit field (with applied RF phases) and the sum-of-magnitude transmit field over the regions-of-interest. Transmit field (B_1_^+^) maps were measured using the SA2RAGE sequence [37] and normalized to 1 kW delivered RF power at the coil plug to assess the efficiency of the optimized RF phases. The B_1_^+^ field value averaged over the left/right inner-ear area was calculated for each of the subjects to evaluate the variability across them. For a single shimmed region, the whole optimization process, including the 3D B_1_^+^ map acquisition, could be performed in less than 5 minutes. Safe RF power limits were derived from 10-gram tissue-averaged Q-matrices [38] computed for the worst-case scenario, that is defined as the RF phase combination producing the highest achievable SAR_10g_ levels. The worst-case computation was derived from electromagnetic field simulations (Sim4Life 4.2, ZMT, Switzerland) using the exact model of the coil array and a realistic human model, Duke [39].

### Single-transmit mode

Single-transmit MR measurements were performed using a commercial 1Tx/32Rx RF coil (Nova Medical, Wilmington, MA, USA). To enhance the transmit field efficiency in temporal lobes and inner-ears, dielectric pads (7TLF Pads of 21.5 cm x 16 cm, Multiwave Innovation, Marseille, France) were used. They were placed on each side of the subjects’ heads (left and right), near to inner-ears expected position. B_1_^+^ maps were measured with and without (for one subject only) the dielectric pads to assess the gain of transmit field efficiency in the regions-of-interest. The results were compared to the pTx coil array.

### MR acquisitions

The following MR sequences were used in both single transmit and pTx modes with the corresponding commercial 1Tx/32Rx RF coil and the 8Tx/32Rx dipole coil array.

Structural MR images of the inner-ears were acquired with a 3D constructive interference in steady-state (3D-CISS; TR/TE = 5.66/2.5 ms, resolution = 0.4 mm-isotropic, FA = 50°, FOV = 128 x 128 x 48 mm^3^, TA = 3 min 35 s) [40]. The sequence was used as it was shown to perform efficiently for cranial nerve and inner-ear imaging [40]. A 3D turbo-spin echo sequence was also used (3D-TSE, TR/TE = 2000/118 ms, resolution = 0.5 mm-iso and 0.8 mm-iso, FA = 120°, FOV = 192 x 192 x 144 mm^3^, GRAPPA = 4, TA (0.5 mm) = 12 min, TA (0.8 mm) = 10 min 40 s). For both measurements, the same optimal RF weights were used in pTx mode. The RF power limits were defined in pTx mode with respect to the worst-case scenario that corresponds to the RF weights producing the highest possible SAR_10g_ value. Therefore, while the calibrated voltage was about 110V, the 3D-TSE was acquired at 60V for the 0.5 mm isotropic resolution and 90V for the 0.8 mm. The 3D-CISS images were acquired at 35V.

**Fig 2:**
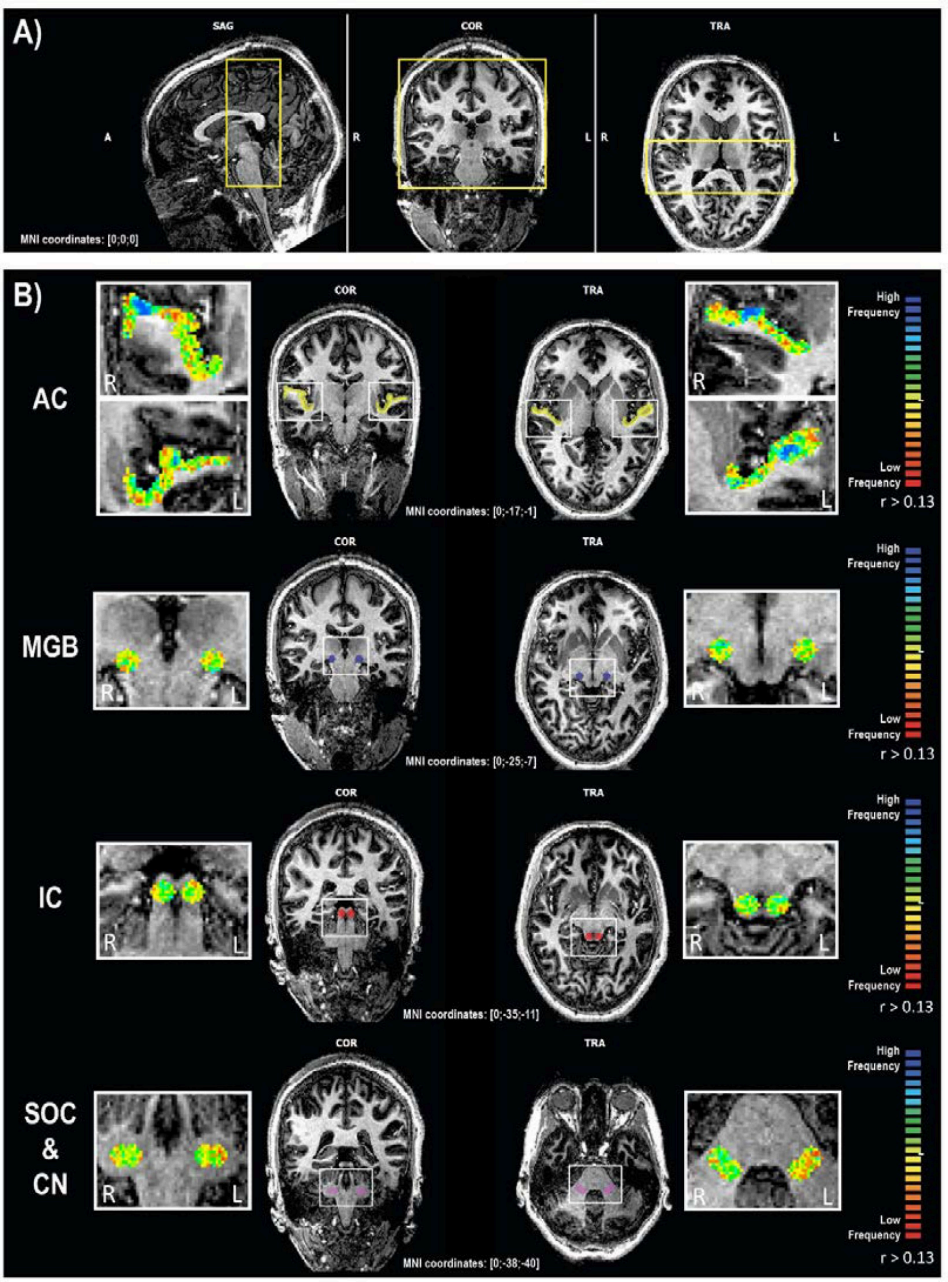
fMRI acquisition along the auditory pathway. A) fMRI slab acquisition (yellow box) projected onto the MP2RAGE; B) fMRI activations reflecting gradients of frequency preferences within the ROIs of the auditory pathway. Tonotopic maps were projected onto the MP2RAGE and masked by the ROIs. The second and third columns illustrate the localization of the ROIs in the coronal and transversal view. The threshold of the correlation values was set to r > 0.13 for visualization purposes only. Voxels preferring low frequencies are represented in red, while those preferring high frequencies are in blue. SAG: Sagittal; COR: Coronal; TRA: Transverse; A: Anterior; P: Posterior; R: Right; L: Left; AC: Auditory cortex; MGB: Medial geniculate bodies; IC: Inferior colliculi; SOC & CN: Superior olivary complex and cochlear nuclei.

The fMRI images of all the auditory relays were acquired simultaneously with a coronal 2D-EPI sequence (TR/TE = 2000/24 ms, resolution = 1.2 mm-iso, FA = 90°, FOV = 180 x 180 mm^2^, GRAPPA = 3, 80 volumes, 43 slices, TA = 2 min 40s, phase-encoding direction: foot-head, total of 6 runs). The field-of-view covered fully all subcortical regions and partially the auditory cortices (yellow box in Fig 2A). The resolution of 1.2mm-iso was chosen to sufficiently sample all the subcortical regions with enough voxels as they have a width of 4-5mm, thus the coverage was of ~ 3.3 voxels, compared to ~ 2.6 in the case of a coarser resolution (i.e. 1.5 mm-iso). Moreover, this voxel resolution was selected in accordance to previous fMRI studies at 7 Tesla which sampled the same ROIs separately [1–3]. After visual inspection, our results were comparable to results of the hereinabove mentioned studies. T1-weighted MP2RAGE images (TR = 6000 ms, TE = 3.03 ms, TI1/TI2 = 800/2700 ms, FA1/FA2 = 7°/5°, resolution = 0.6 mm-iso, 256 slices, TA = 10 min, FOV = 192 x 192 mm^2^) were acquired for alignment of the functional maps to the human brain structures.

### Auditory stimuli

The auditory stimuli were presented bilaterally to the subject using MR-compatible in-ear headphones (SensiMetrics S14, SensiMetrics, USA) and consisted of four ordered progressions of pure tone bursts of 2s each with central frequencies ranging from 88 Hz to 8 kHz, in half octave steps. Each cycle was designed as a block of 14 frequencies (28 s of sound) followed by a “silent” break of 12 s (thus a block length of 40 s) repeated four times (total length of the paradigm was 2 min and 40 s). This paradigm was adapted from the paradigm used in Da Costa et al. [6] (see Fig 1A for the detailed diagram of each cycle), with the same frequency range but longer “silent” breaks.

### Data processing

The right inner-ear was manually segmented from the 3D-CISS images using the MITK (German Cancer Research Center, Heidelberg, Germany) editing tools in the segmentation panel to manually select the ROI and 3D reconstructed it to evaluate the achievable coverage of the region.

fMRI acquisitions were processed with BrainVoyager 20.6.2 (BVQX 3.6.2, Brain Innovation, Maastricht, Netherlands), using the following steps: (1) temporal filtering (with an high-pass filter), (2) motion detection and correction (linear and sinc interpolation respectively), (3) normalization of the fMRI data to the subject corresponding MP2RAGE in the MNI space, (4) linear cross-correlation analysis of the fMRI time courses in the MNI space in order to define frequency preferences for each voxel in a winner-take-it-all approach (as in [5,6,9,41]), and (5) one-sample t-tests on the cross-correlation maps across all subjects within Matlab. Functional data was not smoothed during preprocessing, neither the resulting cross-correlation maps. The six resulting correlation maps (one per functional run) were averaged together and projected onto the individual volumetric MNI space [6]. The averaged map was masked with regions of interest (ROI) defined using the drawing tools of BrainVoyager 20.6.2, as spheres (radius = 4 mm) centered in the structures of interest which were selected based on the online version of the human brain atlas (www.thehumanbrain.info/brain/sections.php; Fig 2B: cochlear nuclei & superior olivary complexes in pink, medial geniculate bodies in blue, inferior colliculi in red, and Table 1). However, due to the larger radius size compared to the relatively small size of the structures, the ROI for the cochlear nuclei and superior olivary complex may also include other non-auditory relays and should not be considered as an “only auditory” ROI.

**Table 1:** MNI coordinates of the ROIs.

	MNI coordinates [X; Y; Z]	Number of voxels
*Right hemisphere*		
inferior colliculi RH	[5; -35; -11]	257
medial geniculate bodies RH	[-14; -25; -7]	257
cochlear nuclei & superior olivary complex RH	[15; -38; -40]	485
*Left hemisphere*		
inferior colliculi LH	[-5; -35; -11]	257
medial geniculate bodies LH	[14; -25; -7]	257
cochlear nuclei & superior olivary complex LH	[-14; -38; -40]	485

## Results

### High-resolution structural MR imaging

The transmit field efficiency measured in the subcortical regions of the brain for the optimized RF phases in pTx mode (Fig 1A) resulted in a mean B_1_^+^ value of 8.9 ± 0.6 μT/√kW over the inner-ear area, with the ± referring to the standard deviation. The mean B_1_^+^ values achieved in single transmit mode over the same region were 38% and 17% lower, without and with the dielectric pads, respectively (Fig 1B and 1C). A mean B_1_^+^ value of 8.4 μT/√kW was achieved in pTx mode across the nine subjects with a standard deviation of 0.7 μT/√kW.

**Fig 3:**
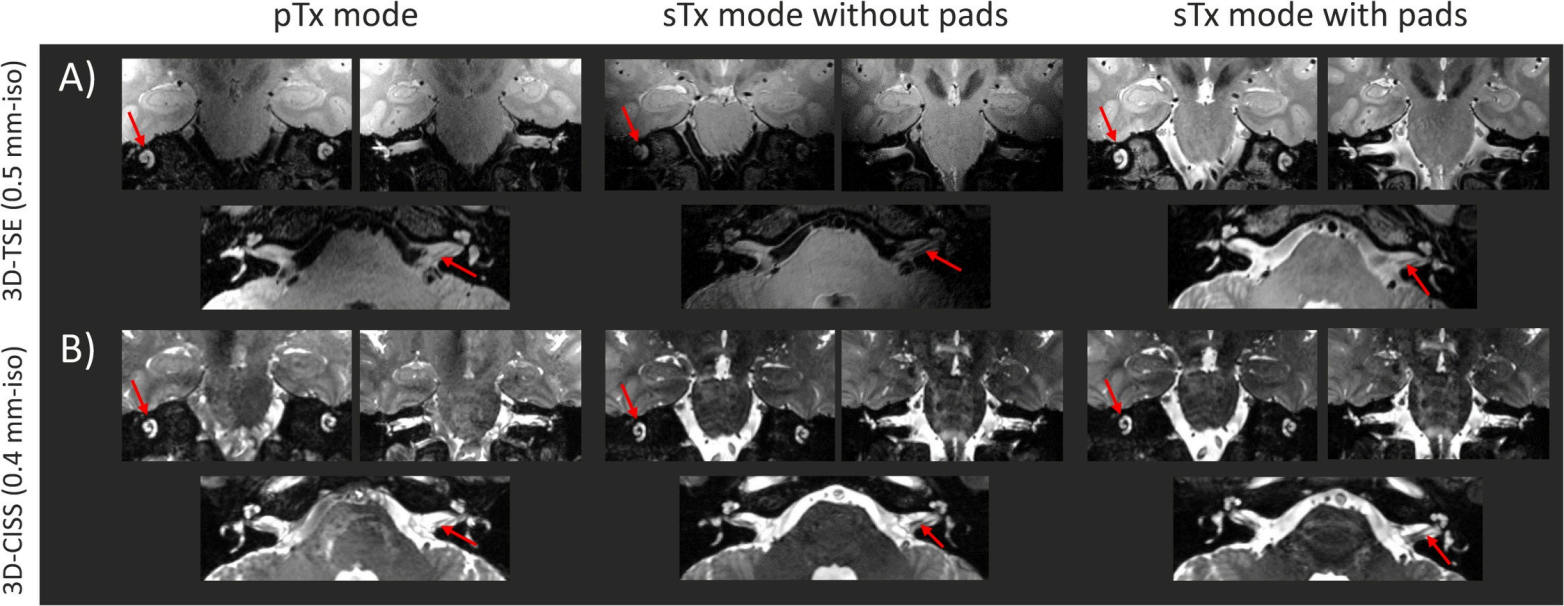
High-resolution MR images of the inner-ears, given for the coil models shown in Fig 1. For parallel-transmit (pTx mode), and single transmit modes with and without dielectric pads (sTx mode without/with pads, respectively): A) high-resolution 3D-TSE (0.5 mm-isotropic) and B) high-resolution 3D-CISS (0.4 mm-isotropic) images shown in two coronal and one transverse slice. The red arrows indicate the structures of interest, namely the cochlea and the auditory nerves. The MR images are shown for a same subject.

With both 3D-TSE and 3D-CISS sequences, a clear visualization of the left and right inner-ears was achieved in pTx and single transmit modes using the optimal RF weights, and dielectric pads, respectively (Fig 3). The 3D-TSE images acquired in single transmit mode without dielectric pads demonstrated a clear signal drop on each side, and the inner-ears were difficult to discern. However, with the 3D-CISS sequence, due to the contrast-nature of the protocol (based on maximum intensity projection of successive phase cycles) the signal was improved and both inner-ears were discernible.

**Fig 4:**
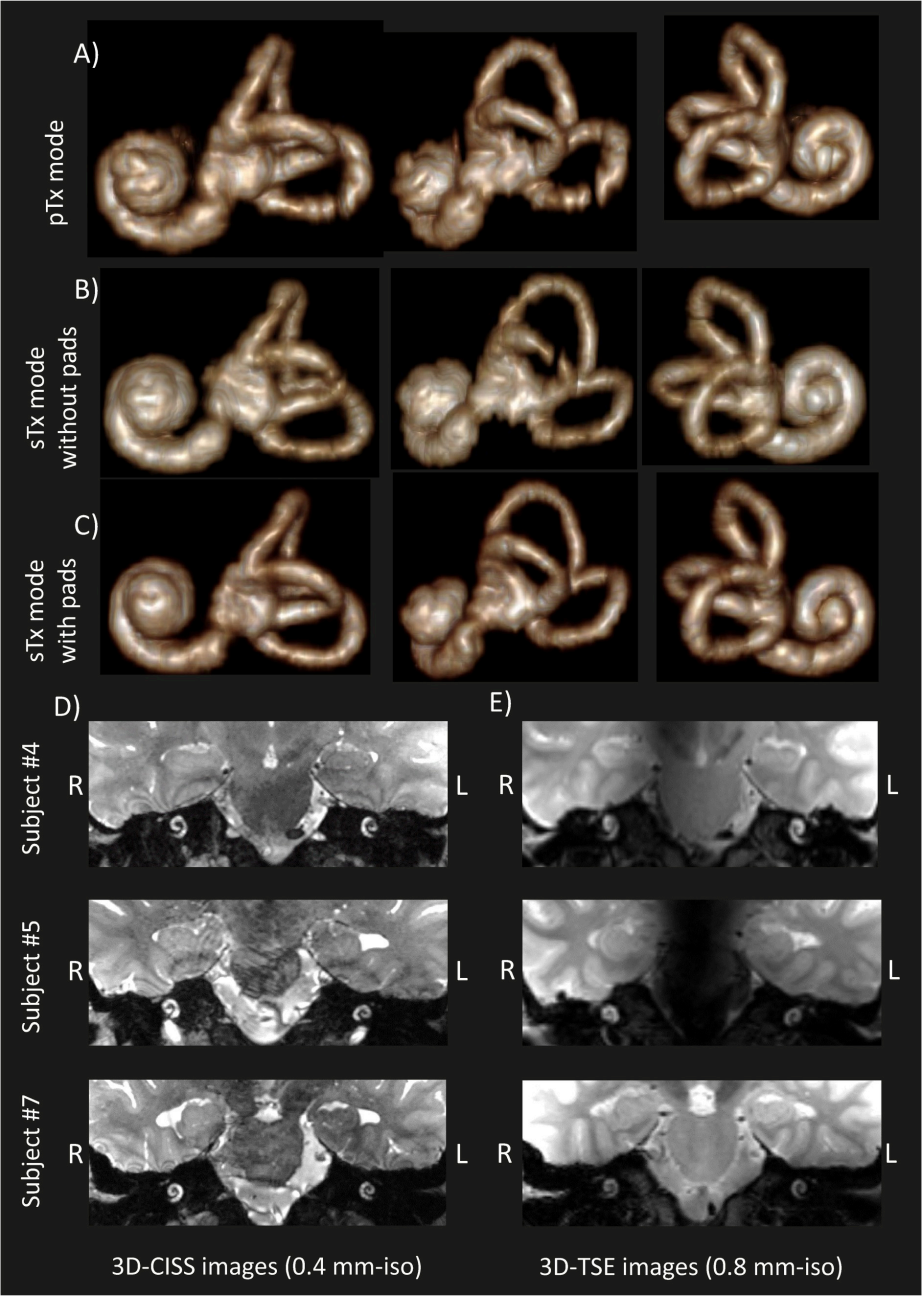
Constructed 3D volume renderings of the left inner-ear for a same subject. Constructed 3D volume renderings of the left inner-ear for a same subject in: A) pTx mode and B) & C) sTx mode without and with dielectric pads, respectively. The segmentation was performed manually using the high-resolution (0.4 mm-isotropic) 3D-CISS images shown in Fig 3B. The cochlea and vestibular system can be clearly identified. For parallel-transmit mode D) high-resolution 3D-CISS (0.4 mm-isotropic) and E) high-resolution 3D-TSE (0.8 mm-isotropic) images of the left and right cochlea shown in coronal plane for three representative subjects (different from Fig 3A–3C).

The complete coverage of the inner-ear was assessed with the 3D reconstruction of the left inner-ear (Fig 4A–4C) where the cochlea, vestibule and semi-circular ducts could be clearly identified. Nevertheless, in both pTx and single transmit modes without dielectric pads one of the semi-circular ducts presented a signal drop.

Comparable results were observed across different subjects using the pTx methods with a clear distinction of the two inner-ears when using either 3D-CISS or 3D-TSE (Fig 4D and 4E).

### High resolution functional MR imaging

**Fig 5:**
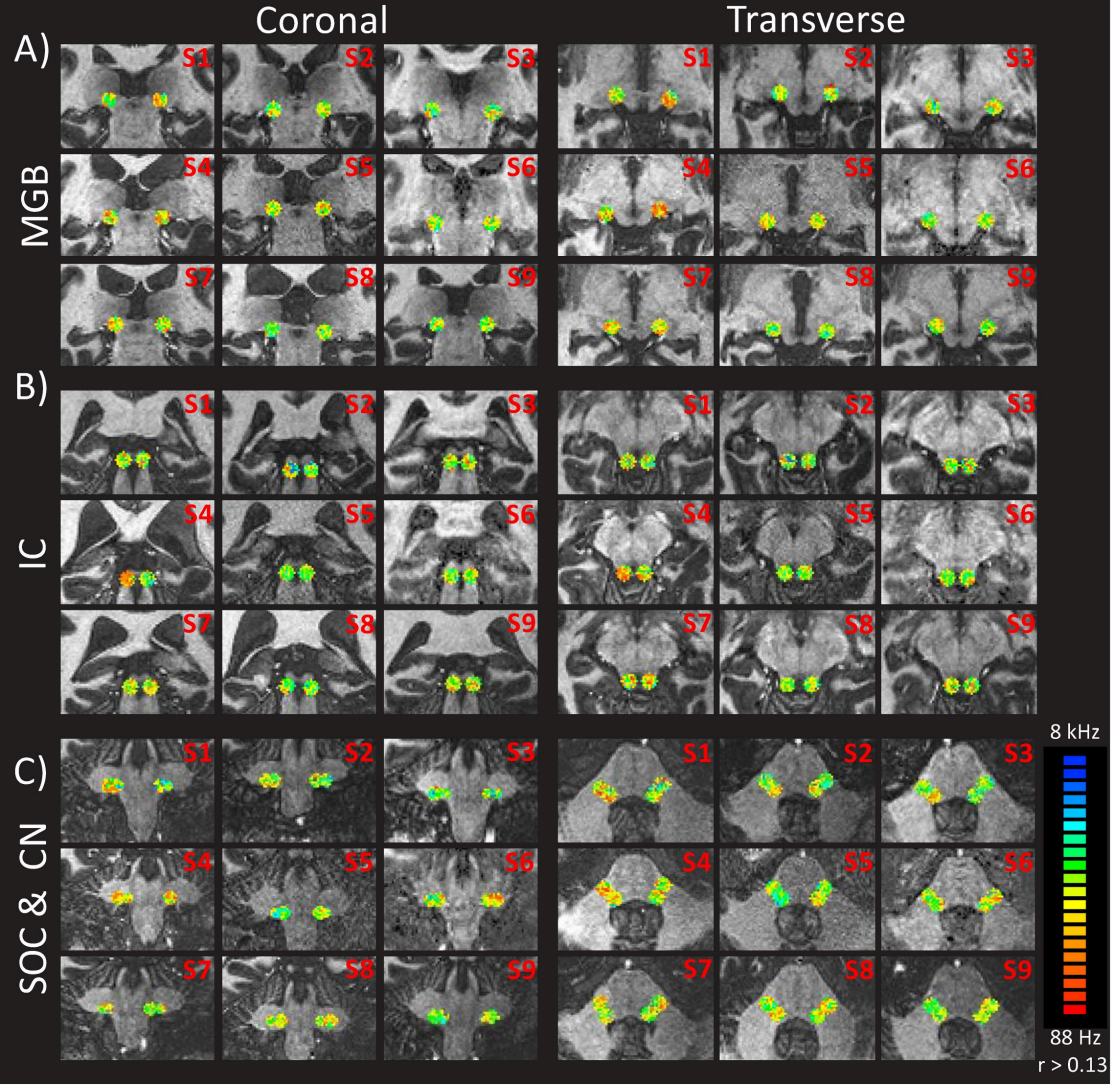
fMRI activations reflecting gradient of frequency preferences within the ROIs of A) the medial-geniculate bodies, B) the inferior-colliculus and C) the superior olivary complex and cochlear nuclei. The coronal and transverse maps are shown for all the subjects (subject number at top right corner). The threshold of the correlation values was set to r > 0.13 for visualization purposes only. Voxels preferring low frequencies are represented in red, while those preferring high frequencies are in blue.

To facilitate the visualization of all frequency gradients the tonotopic maps were projected onto the structural image at the same correlation value (r > 0.13, uncorrected) for each ROI, which revealed frequency gradients in all ROIs (Fig 2B). At the level of the auditory cortices, frequency gradients were ordered bilaterally in a V-shape organization with two mirror-symmetric representations, with voxels preferring low frequencies localized in the crow of the Heschl’s gyrus and voxels preferring high frequencies in the banks of the gyrus (Fig 2B, top row). The one-way ANOVA on the frequency preferences revealed a significant difference between subjects (P_sub_(F>36.83) = 4.3*10^−57^), but no differences across frequencies (P_freq_(F>1.05) = 0.2357). In the medial geniculate bodies, tonotopic representations were structured in a V-shaped low-high-low frequency gradient, organized along a superior-medial to an inferior-central back to a superior-lateral axis (Fig 2B, second row; and Fig 5A). The one-way ANOVA highlighted again a significant difference between subjects but not between frequencies (P_sub_(F>69.9) = 6.2*10^−109^); P_freq_(F>1.06) = 0.1648). In the inferior colliculus, the frequency gradients were organized in a semi-circular low-high-low frequency progression along an inferior-lateral to superior-medial axis (Fig 2B, third row; and Fig 5B). Finally, at the level of the cochlear nuclei & superior olivary complexes, the frequency gradients appeared to be partially organized along two consecutive low-high-low-high gradients arranged along a posterior-lateral to an anterior-medial axis (Fig 2B, last row; and Fig 5C). The one-way ANOVA demonstrated significant differences between subjects but not between frequencies (P_sub_ (F>48.8) = 1.14*10^−77^); P_freq_(F>0.88) = 0.9955). Despite some inter-individual variability, the general trend of the frequency gradients was measurable and reproducible in all subjects, depicting specific high-to-low-low-to-high mirror symmetric gradients.

## Discussion

In this study, we demonstrated that the whole left and right auditory pathway was scanned using fMRI and high-resolution anatomical imaging in one scan session of 1-hour length, without moving the subject out. Specifically, we showed optimized RF field in the inner-ear region and the auditory cortices for high-resolution anatomical MRI, and evaluation of simultaneous functional responses along the left and right auditory pathway, using pTx. Our protocol allow for the functional assessment of the major auditory relays, including cochlear nuclei and the superior olivary complexes, simultaneously within the same volume of acquisition.

The transmit-field efficiency in the inner-ears after RF phase optimization in pTx mode was higher compared to single transmit mode with or without dielectric pads. The increased transmit field was assessed across the subjects, which demonstrates the robustness of the pTx approach to provide high B_1_^+^ field levels in the lower regions of the brain. However, the obtained increased transmit field efficiency was not sufficient using pTx, since the effective flip angle was not achieved due to the worst-case local SAR limits. The safe operation RF limits can in the future be further improved by making use of the Virtual Observation Points (VOP) method to compute in real-time the local SAR_10g_ levels associated to the optimized RF phases [42].

High-resolution imaging of the cochlear and vestibular structures is immensely clinically relevant, for example, in pre-operative imaging when otorhinolaryngology surgeons have issues judging the endolymphatic hydrops within the vestibular structure in Menière’s disease or assessing the length and shape of the cochlea prior to cochlear implant surgery. In our study, we demonstrated a full coverage of the inner-ear structures with high-resolution anatomical images, using parallel-transmit mode as well as single transmit mode only when dielectric pads were used (Fig 4). Without dielectric pads, low signal was observed in the 3D-TSE images. Small signal voids were observed in the 3D-reconstruction of the inner-ear when using pTx, and single transmit mode without dielectric pads. However, pTx allows optimization of different anatomical regions in one session. On the other hand, using dielectric pads only allows transmit field enhancement in one anatomical region once they are placed. To scan different auditory regions, the dielectric pad position needs to be displaced towards the aimed region. This step requires moving the subject.

Applying dielectric pads significantly improved the transmit field efficiency in single transmit mode in the region-of-interest. However, placement of the pads was critical to achieve the targeted improvements and can be unpractical to use as they hardly fit within the RF coils and mechanical deformation of the pads can alter their efficiency [43]. Moreover, dielectric pads did not yield transmit field enhancement at the central base of the brain. Therefore, they were unsuitable to improve the RF signal in the subcortical regions of the auditory pathway. RF phase optimization in parallel-transmit mode was performed in 5 minutes, which lengthen reasonably the scan time while providing higher transmit field performances in the region-of-interest compared to using dielectric pads.

Functional cross-correlation maps highlighted tonotopic representations along all relays of the auditory pathway with clear or partially clear frequency progressions, similar to previous studies (cochlear nuclei: [1]; inferior colliculi: [2]; medial geniculate bodies: [3,44]; auditory cortices: [4–7]). The present study revealed several differences in the axis of frequency gradients at the sub-cortical level (Figs 2 and 5) which might be due to the use of pure tones instead of natural sounds and/or of larger, less specific ROIs (compared to [1,2,44]). Note that the functional results presented here are only descriptive, as the scope of this study was to evaluate the functional activation within all relays simultaneously using the pTx system and not their individual frequency mappings. Nevertheless, the results indicate potential for clinical applications as functional activations of the auditory pathway in less than 12 min were measured, compared to classical fMRI sessions ranging from 24 min [11] to ~80 min [3]. Further studies will have to (a) adapt the MR sequences to match SAR requirements, (b) improve the anatomical localization of the subcortical regions using finer alignments with post mortem data or with functional atlases from other studies, (c) acquire a larger group of healthy subjects to quantify individual variability with specific analysis, and (d) compare the results between different auditory stimuli (pure tones vs. natural sounds) and different clinical populations.

The fMRI protocol yielded reproducible and consistent results across subjects with specific high-to-low-low-to-high mirror symmetric gradients at the level of the auditory relays (Fig 5, [4–7]). However, frequency preferences were less clear than in previously findings. In our study, the target region for RF shimming was essentially covering the subcortical areas instead of cortical ones, thus resulting in less specific cortical mappings as the auditory cortices were more susceptible to suffer from subject’s motion or vessel artefacts compared to previous studies exclusively tuned for the superior temporal gyrus. Results at the level of the cochlear nuclei were less consistent and specific, and thus were not comparable to results from the animal literature. Future studies should investigate such comparison using MR sequences with a field of view focusing only on these nuclei instead of a larger slab covering as much as possible the auditory pathway.

Within a standard 1-hour scan session, the transmit field optimization followed by the high-resolution MR acquisition (3D-TSE and 3D-CISS) takes approximately 30 min. Functional data was then acquired and reconstructed for 20 min, and clearly demonstrated frequency preferences at all levels of the auditory pathway. Standard protocols achieved similar functional results in the medial geniculate bodies, inferior colliculi and auditory cortices separately with a session of approximately 90 min and 60 min, respectively [5,6]. Our approach reduced the scan time by a factor of 2.5 and was performed within a single session, making it more tolerable for clinical populations.

## Conclusion

We conclude that at 7T complete coverage of the inner-ear can be achieved on healthy subjects using optimized RF transmit field in pTx mode and may be used in the future to image structural lesions in patients suffering from hearing loss. This was combined with functional tonotopic maps to assess functional reorganization, yielding a complete evaluation of the auditory pathway with scan time compatible with clinical examination.

## Supporting information

S1 File (ZIP)

S2 File (ZIP)

S3 File (ZIP)

S4 File (ZIP)
